# Effects of Sequential Antimicrobial Phases on Root Canal Microbiome Dynamics in Two-Visit Treatment of Primary Apical Periodontitis: A Longitudinal Experimental Study

**DOI:** 10.3390/life14121696

**Published:** 2024-12-21

**Authors:** Bertan Kesim, Seda Tezcan Ülger, Gönül Aslan, Yakup Üstün, Ayşe Tuğba Avcı, Mustafa Öner Küçük

**Affiliations:** 1 Department of Endodontics, Faculty of Dentistry, Nuh Naci Yazgan University, Kayseri 38170, Turkey; 2 Department of Medical Microbiology, Faculty of Medicine, Mersin University, Mersin 33343, Turkey; 3 Department of Endodontics, Faculty of Dentistry, Erciyes University, Kayseri 38039, Turkey; 4 Department of Microbiology, Faculty of Dentistry, Nuh Naci Yazgan University, Kayseri 38170, Turkey

**Keywords:** 16S ribosomal RNA sequencing, calcium hydroxide, antimicrobial procedures, microbiome modulation, 2-visit root canal treatment, *Bifidobacterium longum*

## Abstract

Background: Effective management of primary apical periodontitis depends on understanding the dynamic interactions within the root canal microbiome. This study aimed to investigate the effect of sequential antimicrobial phases on the root canal microbiome during a two-visit treatment approach, with a focus on calcium hydroxide medication. Methods: Samples were collected from three teeth across four treatment phases: initial infection (S1), after chemomechanical preparation (S2), after intracanal medication (S3), and after a final flush (S4). DNA was extracted, and the V3–V4 regions of the 16S rRNA gene were sequenced using Illumina MiSeq. Sequencing data were analyzed with QIIME 2, and differentially abundant taxa were identified using linear discriminant analysis effect size (LEfSe). Results: While microbial community composition did not differ significantly between phases, the *Firmicutes*/*Bacteroidetes* ratio decreased after the antimicrobial stages. LEfSe analysis revealed higher abundances of *Lactobacillales*, *Arthrobacter*, and *Veillonella* in the untreated (CMP) group. *Bifidobacterium longum* was relatively more abundant in the intracanal medication (ICM) phase, and *Dorea formicigenerans* was more abundant in the final-flush (FF) phase. Conclusions: Although calcium hydroxide treatment did not induce statistically significant changes in overall root canal microbial composition, trends such as a reduction in the *Firmicutes*/*Bacteroidetes* ratio and a relative increase in *Bifidobacterium longum* numbers suggest potential ecological shifts. The observed relative increase in *Bifidobacterium longum* numbers may represent a hypothesis-driven observation reflecting indirect ecological effects rather than direct pH modulation. While visual patterns (e.g., PCA clustering) were observed, they lacked statistical support. Further studies with larger sample sizes are needed to validate these observations and assess the potential role of beneficial bacteria in root canal treatments.

## 1. Background

Apical periodontitis is an inflammatory process triggered by microorganisms invading the root canals and leading to bone damage around the root apex [1]. The eradication of microorganisms from the root canals leads to the resolution of osteopathy in the periapical region and promotes bone healing [2]. To ensure root canal treatment (RCT) is successful and apical periodontitis is cured, it is essential to lower the bacterial load in the root canals below a certain threshold [3] and eliminate highly pathogenic microorganisms in the root canals.

Ongoing research is aiming to establish the most effective approach for disinfecting root canals, which remains a challenging process. Chemomechanical preparation plays the most significant role in removing bacteria from root canals [3]; nevertheless, there may be situations in which additional disinfecting actions via root canal medications are needed. High levels of disinfection may be required in cases with unfavorable prognostic factors before treatment, and in symptomatic cases, a clinician may choose to visually verify the disappearance of symptoms before completing treatment. Due to its antimicrobial properties [4], calcium hydroxide is widely used in intracanal therapy to counteract the inflammatory effects of bacterial substances [5,6] and prevent bone degradation [7].

The success rate of RCT can be influenced by the use of calcium hydroxide medication in the root canals and the number of visits involved [8]. There are different perspectives and conflicting reports in the literature on the antimicrobial properties of calcium hydroxide and how this medication affects the outcomes of endodontic treatments. In an earlier report [9], it was noted that leaving calcium hydroxide in root canals for at least one week led to negative cultures in 92.5% of the canals. In a subsequent study, Siqueira et al. showed that using a calcium hydroxide dressing boosted the antibacterial impact of chemomechanical treatment with 2.5% NaOCl [10]. However, it is clear that calcium hydroxide does not yield consistent results against all microorganisms [11]. Furthermore, a recent meta-analysis [12] indicated that single-visit RCT had superior treatment success rates compared to multiple visits.

Pioneering molecular studies have shown that calcium hydroxide medication reduces the bacterial load and bacterial diversity in root canals [3,13,14]. Root canal infections are isolated, mostly anaerobic infections triggered by complex microbial profiles [15]. High-throughput next-generation sequencing (NGS) platforms have become the preferred method for exploring diverse microbial ecosystems [16]. A sequence of independent disinfection steps is involved in RCT, which can be implemented with different protocols. Determining which organisms are susceptible or resistant to sequential antimicrobial phases is crucial for developing next-generation antimicrobial protocols. Current disinfection strategies, while effective to some extent, often fail to address the complex and resilient microbial ecosystems in root canal infections.

Louzada et al.’s molecular research [17] provided quantitative evidence of the impact of calcium hydroxide medication on reducing the bacterial products responsible for root canal infections; their results were consistent with earlier findings [5,6]. Moreover, Erşahan et al. confirmed there was a contribution of calcium hydroxide medication to reducing the total bacterial load in the root canals of teeth with apical periodontitis after treatment utilizing droplet digital PCR (ddPCR) [18]. While the antimicrobial properties of calcium hydroxide have been previously demonstrated, its specific impact on the root canal microbiome, particularly in terms of microbial diversity and composition during sequential antimicrobial phases of treatment for primary apical periodontitis, remains unclear. In this study, we employed next-generation sequencing to address this gap and provide deeper insights into microbiome dynamics in the context of endodontic therapy. A comparison was made with different disinfection procedures via a two-visit strategy.

This study aims to investigate the dynamic changes in the root canal microbiome during sequential antimicrobial phases of a two-visit treatment for primary apical periodontitis, with an emphasis on microbial diversity and resistance patterns. The null hypothesis of this study was that treating root canals with calcium hydroxide for 1–2 weeks, along with other disinfection procedures, would not alter bacterial composition and diversity in the root canal.

## 2. Methods

Prior to this study’s initiation, approval was obtained from the Erciyes University Clinical Research Ethics Committee (decision no. 2022/752), and root canal samples were obtained exclusively from volunteers who had signed informed consent forms. This study followed the ethical standards outlined in the 1964 Declaration of Helsinki and its subsequent amendments. Three patients who sought treatment for primary apical periodontitis at the Erciyes University Faculty of Dentistry’s Endodontics Clinic were included in this study, for which a clinical trial number is not applicable. This study adhered to the STROBE (Strengthening the Reporting of Observational Studies in Epidemiology) guidelines for observational studies.

*Inclusion Criteria*

The inclusion criteria for this study were as follows:

Healthy individuals aged 18–65 years with no significant medical history.
Individuals who had not used antibiotics in the last 3 months.
Only one tooth per patient was sampled.
Patients with symptomatic mandibular molar teeth with the following characteristics:
  - Periapical lesions ≤ 3 mm.
  - A pre-procedure Periapical Index (PAI) score of 2, 3, or 4 [19].
  - Not subjected to previous root canal treatment (RCT).

*Exclusion Criteria*

The exclusion criteria for this study were established under 2 sub-headings to minimize confounding factors and ensure reliable microbial analysis.

Individuals with the following systemic conditions:

- Diabetes mellitus.
- Autoimmune diseases.
- Compromised immune systems.
- Cancer or other chronic illnesses.
- Pregnant or breastfeeding women.

Teeth with the following characteristics:

- Unsuitability for rubber dam isolation.
- Exposed pulp chambers or root fractures.
- Sinus tract, swelling, or deep periodontal pockets (>4 mm).
- Open apex or root resorption.
- Placement under bridges or crowns.
- History of previous root canal treatment (RCT).

Periapical X-rays of the patients’ mandibular molar teeth were taken using the long-cone paralleling technique with phosphor plates. Two observers examined all radiographs (Y.Ü. and A.T.A.). PAI observer calibration, which was carried out according to the method reported by Ørstavik et al. [19], led to a Cohen’s Kappa score of 0.85. Before sampling, data on demographic factors, such as age and gender, as well as the type and quality of previous dental restorations of the teeth were documented.

### 2.1. Microbial Sampling of Root Canals

Strict aseptic procedures were followed during root canal sampling, as described previously [20]. The study volunteers were directed to rinse their mouths using a 1% H_2_O_2_ solution. Each tooth was initially cleaned with pumice and isolated with a rubber dam. A meticulous process of decontamination was carried out on the exterior of the teeth, involving the application of 3% H_2_O_2_, followed by 2.5% sodium hypochlorite (NaOCl). Under local anesthesia (4% articaine with 1,200,000 epinephrine), a high-speed diamond bur cooled by water was used to remove caries and access the pulp chamber. A repeated disinfection process was applied to the pulp chamber and access cavity walls. To neutralize the disinfectants and prevent any lingering effects on root canal sampling, 5% sodium thiosulfate was used. Sterility control samples were collected to confirm the effectiveness of the disinfection process and ensure the validity of the microbiome analysis. These samples were obtained from the inner walls of the access cavity and the cavosurface angle, representing areas potentially exposed to contamination. The presence of bacteria in the control samples was assessed through PCR [21], but no positive results were found in even one reaction for any of the samples. Distal canal samples from lower molars were utilized for the root canal microbiome analysis [22]. Using a 27-gauge syringe, sterile saline solution was delivered into the root canal, and then an electronic apex locator (Propex Pixi, Dentsply Maillefer, Ballaigues, Switzerland) was used to determine the working length. Samples were taken from the relevant teeth of each patient at four different intervals.

#### 2.1.1. First Visit

Samples S1 and S2 were obtained before and after chemomechanical preparation, respectively, at the first visit. Before the start of the chemomechanical procedures, a sample (S1) was taken from the root canal for use as a point of comparison. Five sterile paper points (size 20, taper 0.02) were placed consecutively along the working length and left in the root canal for 1 min. Chemomechanical preparation was carried out using Reciproc Ni-Ti instruments (VDW GmbH, Munich, Germany). Narrow, medium, and wide canals were shaped using R25, R40, and R50, respectively, based on the canal sizes. After an initial flush with 10 mL of NaOCl (2.5%), the cervical, middle, and apical thirds of the root canal were prepared sequentially. Each step was followed by irrigation with 10 mL of NaOCl (2.5%); a total of 40 mL NaOCl (2.5%) was used until root canal preparation was complete. The root canal was then irrigated with 5 mL of sodium thiosulphate (5%) and filled with sterile saline solution before sampling after instrumentation was selected (S2). Then, five sterile paper points (ISO 20, taper 0.02) were sequentially placed at the working length, and each paper point was left in the root canal for 1 min. UltraCal XS calcium hydroxide paste (Ultradent Products, South Jordan, UT, USA) was placed in the root canals at working length using a 29-gauge (0.33 mm) NaviTip and delivered into the canal until it reached the level of the canal orifice. The access cavities were sealed with conventional glass ionomer cement (MeronPlus, Voco GmbH, Cuxhaven, Germany) that was a minimum of 2 mm thick. The root canals were filled with calcium hydroxide paste for a period of 1–2 weeks. Although prolonged use of calcium hydroxide may weaken dentin [23], a 1–2-week duration was chosen to ensure optimal antimicrobial activity [24].

#### 2.1.2. Second Visit

Samples of S3 and S4 were collected during the second visit right after the intracanal medication had been removed and before the root canal was filled after the final irrigation. After 1–2 weeks, the teeth were isolated, the temporary restorations were removed, and the operative area was disinfected following the same protocol used in the first visit. A new control sample was obtained from the walls of the access cavity surrounding the pulp chamber. The calcium hydroxide paste was removed from the canal via agitation with 10 mL of ethylenediaminetetraacetic acid (EDTA) (17%) and K-type files, and a third sample was taken after irrigation with 5 mL of sterile saline solution (S3). As in the previous samples, five sterile paper points (ISO 20, taper 0.02) were placed consecutively at a working length, and each was left in the root canal for 1 min. The root canal was irrigated with 10 mL of NaOCl (2.5%), and an inventory of instrumentation was made with a Reciproc file of the same size as the one chosen during the first visit. Final irrigation with 10 mL of NaOCl (2.5%) was followed by final neutralization with 5 mL of sodium thiosulfate (5%). After instrumentation, the root canal was filled with sterile saline before sampling. At the end of the procedure, five sterile paper points (ISO 20, taper 0.02) were sequentially placed at working length, following the same protocol applied to the previous groups. Each paper point was left in the root canal for 1 min to collect the S4 samples. After sampling, the root canals were filled using the cold lateral condensation technique, and the access cavities were sealed with composite resin (Z250, 3M Corporation, Saint Paul, MN, USA). The paper points were placed in cryotubes containing 300 μL of RNAlater solution (Life Technologies, Carlsbad, CA, USA) and stored at −80 °C until DNA extraction.

### 2.2. DNA Extraction, PCR Amplification, and Library Construction

The bacterial genetic material from the root canal samples was isolated using the GeneMATRIX Tissue and Bacterial DNA Purification Kit (Roboklon, Berlin, Germany) following the manufacturer’s instructions.

Amplification of the V3–V4 regions of the bacterial 16S rRNA gene was carried out following the Illumina 16S Metagenomic Sequence Library Protocol using specific forward (5′-TCG TCG GCA GCG TCA GAT GTG TAT AAG AGA CAG CCT ACG GGN GGC WGC AG-3′) and reverse (5′-GTC TCG TGG GCT CGG AGA TGT GTA TAA GAG ACA GGA CTA CHV GGG TAT CTA ATC C-3′) primer sequences [25]. The specified amplification process included an initial denaturation at 95 °C for 3 min, followed by 25 cycles of denaturation at 95 °C for 30 s, annealing at 52 °C for 30 s, and elongation at 72 °C for 30 s. The amplification process was finalized with a final extension step of 72 °C for 5 min. The amplified PCR products were separated using electrophoresis in a 2% agarose gel, followed by purification with Agencourt AMPure XP (Beckman Coulter Genomics, Brea, CA, USA). The amplified fragments were sequenced using Illumina MiSeq (Illumina, San Diego, CA, USA), and the sequencing libraries were prepared using a Nextera XT kit (Illumina Inc., San Diego, CA, USA).

### 2.3. Bioinformatic Analysis

The sample size for this study consisted of three teeth per group. This number was determined based on logistical and resource limitations inherent to the study design. A post hoc power analysis was performed, revealing that the statistical power of this study was 46.57% [26]. According to this calculation, a minimum of 26 teeth per group would be required to achieve 80% power [26]. Despite the limited sample size, the sequencing depth (averaging over 2000 reads per sample) provided sufficient coverage for identifying microbial composition and diversity.

Paired-end 250 bp reads (500 cycles) were obtained via sequencing using the Illumina MiSeq system, and paired-end Illumina reads (2 × 250) were then imported into the QIIME 2 environment. Sequences from all samples had depths greater than 100×, and no sequences were flagged as low-quality, resulting in no samples being omitted from the study. Enhancement of read quality, identification of chimeric sequences, and trimming were carried out using the QIIME 2 DADA2 pipeline. Elimination of bases with low phred scores (<Q30) was carried out, and amplicon sequence variants (ASVs) derived from Dada2 were matched to the GreenGenes database (http://greengenes.lbl.gov). Amplicon sequence variants (ASVs) were used instead of operational taxonomic units (OTUs) to provide higher resolution and sensitivity by accurately detecting even single-nucleotide differences, which are often missed when using OTU-based methods [27]. This approach not only enhances the representation of microbial diversity but also improves the detection of rare variants and ensures more reproducible results are obtained across datasets, making ASVs robust tools for microbiome analysis [27]. Phyloseq objects were acquired from QIIME2 files using R 4.1 software. *p*-values were compared between groups using the Kruskal–Wallis test, with a significance threshold set at *p* < 0.05. To address the issue of multiple comparisons, Benjamini–Hochberg (FDR) correction was applied where applicable. The assessment of taxonomic diversity between individuals was determined through beta diversity analysis, utilizing Jaccard, Bray–Curtis, and weighted and unweighted UniFrac metrics. Using the ‘vegan’ R package version 4.1, the statistical significance of alpha diversity (Kruskal–Wallis; *p* < 0.05) and beta diversity (PERMANOVA) was calculated. Differentially abundant bacterial groups at the phylum and genus levels were identified using LEfSe to determine their relevance in various contexts. For LEfSe analysis, a *p*-value threshold of 0.05 and a linear discriminant analysis (LDA) score of >2.0 were set to identify significant biomarkers.

## 3. Results

The MiSeq platform results revealed 2861 ASVs from 12 samples, totaling 1,496,568 sequence reads after ASV filtering, with an average of 124,714 reads per sample. Of these ASVs, 1805 were common among the groups. In total, 1056 different ASVs were distributed among 37 phyla, 95 classes, 150 orders, 207 families, 347 genera, and 220 species. The discrepancies in the ASV numbers between sampling intervals are depicted in Table 1.

**Figure 1:**
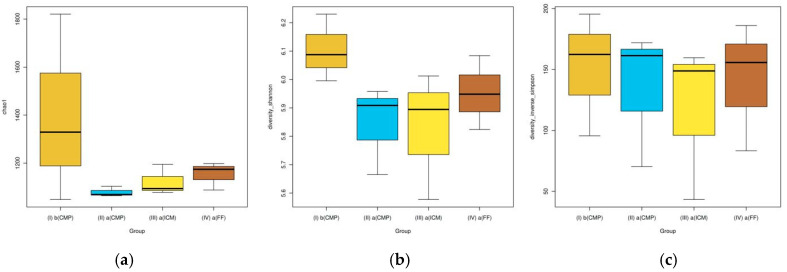
Bacterial diversity indices across study groups. (**a**): The Chao 1 index shows the highest richness in the b(CMP) group (baseline, pre-treatment), indicating a reduction in bacterial diversity following antimicrobial interventions. (**b**): The Shannon index reveals reduced diversity and increased homogeneity in the a(ICM) group compared to other treatment phases. (**c**): The Inverse Simpson index highlights a more balanced and homogenized bacterial composition in the a(ICM) group.

The Chao1 richness index findings indicated no significant disparity among the groups in the alpha diversity evaluation (*p* = 0.70) (Figure 1a). The Shannon and Inverse Simpson plots in Figure 1b,c offer a visualization of community homogeneity, showcasing median and score values.

**Figure 2:**
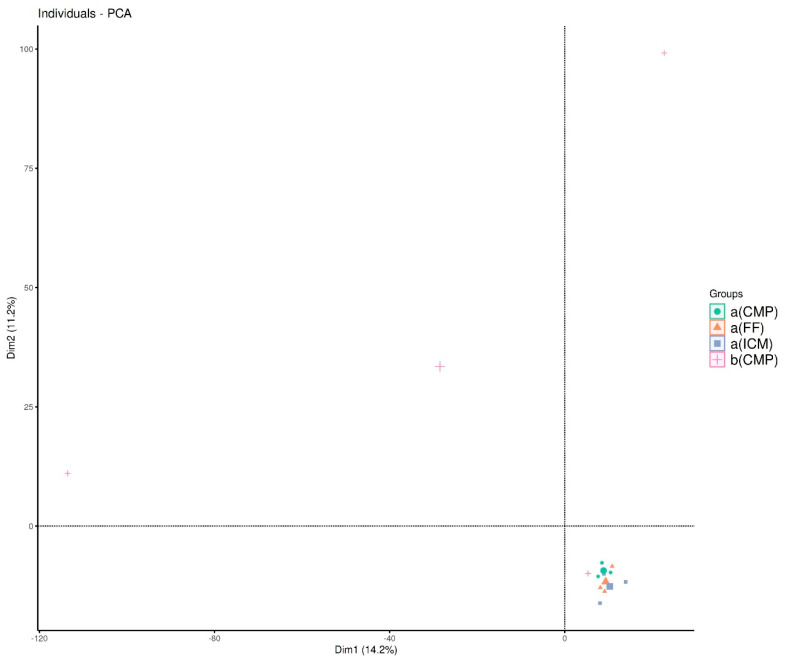
Principal component analysis (PCA) of beta diversity across study groups: The PCA plot shows shifts in bacterial composition throughout treatment phases. The b(CMP) group (baseline) clusters separately from the intervention groups (a(CMP), a(ICM), and a(FF), highlighting changes in microbial community structure after sequential antimicrobial treatments.

Figure 2 demonstrates a visual interpretation of beta diversity obtained via a principal component analysis (PCA).

The PERMANOVA test using Adonis failed to detect a significant effect of the antimicrobial procedures used during RCT on community composition (*r*^2^ = 0.0261, *p* > 0.05). Differences in composition among the groups were examined using the multi-response permutation procedure (MRPP), but no alterations in microbial profiles were found (*A* = −0.039, *p* = 0.96). Although an analysis of similarity (ANOSIM) suggested there were variations between the b(CMP) group and the groups that underwent antimicrobial procedures during endodontic therapy, the typical structures of the microbial populations showed a resemblance with a weak correlation and an insignificant *p*-value (*r*^2^ = 0.145, *p* = 0.181).

**Figure 3:**
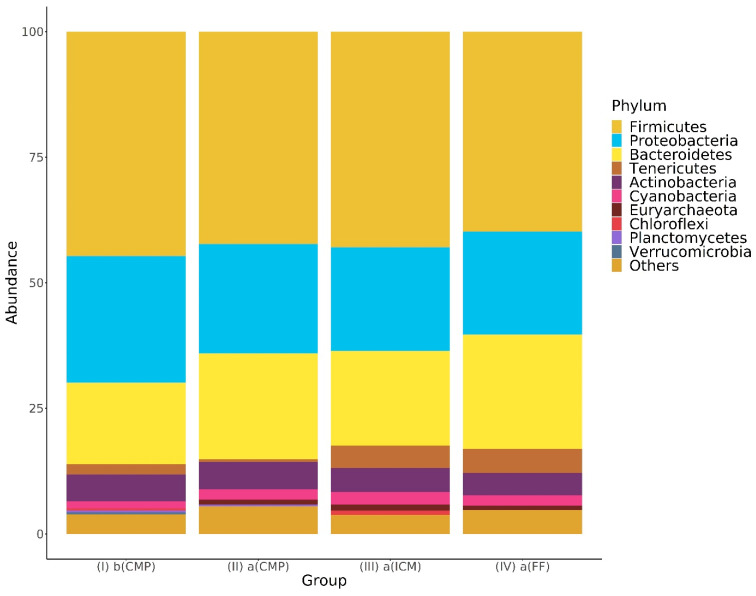
Relative abundance of bacterial phyla across treatment groups: this bar graph shows the changes in bacterial phyla during the treatment phases.

At the phylum level, no taxa were found to have significantly different abundances between groups. In particular, bacterial species belonging to four phyla (*Firmicutes*, *Proteobacteria*, *Bacteriodetes*, and *Actinobacteria*) were the most predominant members of the root canal microbiome of teeth with initial primary apical periodontitis [Group I, b(CMP)] and the microbiome detected during the treatment phases of RCT performed in two sessions (Groups II, III, and IV). The *Firmicutes* proportion notably dropped in Groups II and IV following chemomechanical preparation, with a decrease from 45.64% to 42.11% between Groups I and II and from 42.74% to 38.74% between Groups III and IV (Figure 3).

At the order level, the LEfSe analysis revealed that the b(CMP) group, which was not subjected to any disinfection process, exhibited a significantly greater presence of *Lactobacillales* (Gram-positive +/anaerobic) than the other groups. The family-level abundance of *Methylophilaceae* (Gram-negative/aerobic) was notably greater in the a(FF) group than in the other groups. At the genus level, the lack of disinfection treatment for Group I [b(CMP)] resulted in a statistically significant increase in the relative abundance of *Arthrobacter* (Gram-positive/aerobic) and *Tistrella* (Gram-negative/aerobic). The taxa represented in proportions more than 1% at the genus level and their relative abundance changes between treatment phases are presented in Table 2.

**Figure 4:**
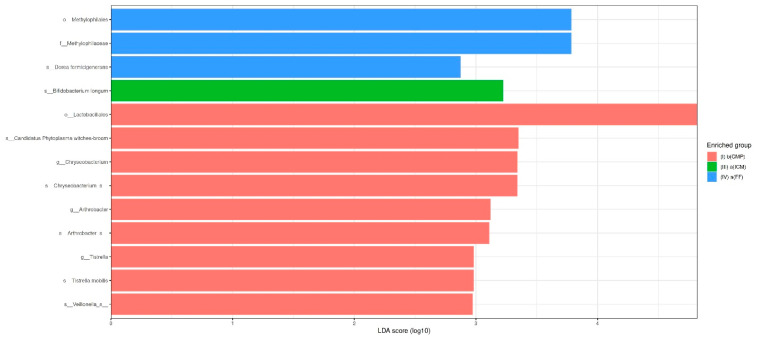
Differentially abundant bacterial taxa are illustrated through horizontal bar graphs. Higher LDA scores reveal group-specific biomarker taxa.

Based on the LEfSe findings, at the species level, Group I [b(CMP)]—the group for which no disinfection interventions were carried out—exhibited a significantly higher relative abundance of *Candidatus Phytoplasma* witches-broom (Gram-positive/*), *Chryseobacterium_s* (Gram-negative/aerobic), *Arthrobacter_s* (Gram-positive/aerobic), *Tistrella mobilis* (Gram-negative/aerobic), and *Veillonella_s* (Gram-negative/anaerobic) than the other groups. In addition, the relative abundances of *Bifidobacterium longum* (Gram-positive/anaerobic) in the a(ICM) group and *Dorea formicigenerans* (Gram-positive/anaerobic) in the a(FF) group were statistically significantly higher than in the other groups. Figure 4 provides a full overview of the findings obtained from the LEfSe analysis.

## 4. Discussion

This study aimed to assess how the root canal microbiome changes during the key antimicrobial stages of two-visit endodontic therapy for teeth with primary apical periodontitis by identifying taxa resistant or susceptible to these disinfection procedures. Few next-generation-sequencing studies [28,29] have evaluated the effects of chemomechanical preparation (CMP) and intracanal medication (ICM) with calcium hydroxide on the microbiomes pertaining to primary endodontic infections. Our study differs from the studies of Kruly [28] and Iriboz et al. [29] in that we conducted a separate evaluation of each phase to determine how CMP, ICM with calcium hydroxide, and final irrigation influence the microbiome, while in the mentioned studies, the cumulative effect of the procedures was evaluated.

In this study, 2.5% NaOCl was used because, in recent years, more moderate concentrations of NaOCl have been reported to have antimicrobial activity similar to full-strength NaOCl [30,31]. A glycerine-containing calcium hydroxide preparation (UltraCal) that has been reported to have high antibacterial activity as a root canal medicament [32] was left in the root canals for 1–2 weeks, constituting the recommended time frame for teeth with primary endodontic infection [33].

The unique sequences obtained were labelled using ASVs rather than OTU clustering in this study. ASV-based techniques reduce sequence errors and can detect biodiversity with higher sensitivity than OTU analysis [34]. In our study, the number of ASVs observed in the initial sampling phase (S1) decreased significantly after the application of calcium hydroxide (S3). Due to calcium hydroxide’s high pH and antimicrobial effects, the authors suggest that applying it to root canals for 1 or 2 weeks may cause a reduction in microbial diversity. To minimize the potential disadvantage of dentin weakening associated with calcium hydroxide [23], the application period was limited to 1–2 weeks, which is generally considered sufficient for allowing its antimicrobial activity to take effect while also avoiding prolonged exposure [24]. The surge in ASVs between S3 and S4 may have been caused by viable or non-viable bacteria spreading through open dentinal tubules following smear layer removal as well as the release of bacteria from the biofilm following CMP and final irrigation.

Alpha diversity is a term used to describe the variety of species within a specific community. In this study, alpha diversity calculations were conducted using the Shannon, Chao 1, observed species, and Simpson indices. Despite the lack of significant differences between groups according to the Chao 1 richness estimator (*p* = 0.7), the highest median Shannon diversity was observed in S1, and the lowest was observed in S3. Moreover, the median value in the S3 sample was found to be lower than the median value in the S2 sample on the inverse Simpson diversity index plot. These results suggest that optimizing the disinfection process by incorporating 1–2 weeks of intracanal calcium hydroxide medication leads to a wider range of antimicrobial effectiveness in root canals than using CMP alone. While calcium hydroxide can effectively restrict bacterial growth, it does not completely eradicate bacteria, as previously reported in the literature [3]. When managing advanced apical periodontitis with extensive lesion sizes, wherein traditional disinfection protocols using NaOCl and calcium hydroxide may not ensure complete bone regeneration, alternative strategies could be considered. For instance, a recent study reported an advanced disinfection protocol that combines calcium hydroxide with copper ions and an electrophoretic current. This approach may enhance antimicrobial efficacy and address resistant microbial taxa more effectively [35]. Such approaches could be beneficial for addressing persistent infections or in cases where conventional methods prove insufficient.

When assessing the similarity or dissimilarity of microbial communities in different sample groups, beta diversity emerged as an important metric. In this study, beta diversity indices were utilized to analyze how similar the bacterial compositions were at various sampling points, specifically during the main antimicrobial phases of RCT. By utilizing a PERMANOVA test along with Adonis, we investigated the influence of antimicrobial methods in RCT on community composition, but no significant effect was detected (*r*^2^ = 0.0261, *p* > 0.05). Group variations were analyzed using the MRPP, revealing no alterations in microbial compositions (*A* = −0.039, *p* = 0.96). The ANOSIM findings revealed group differences, although the microbial populations’ basic compositions exhibited similarity, with minimal correlation and an insignificant *p*-value (*r*^2^ = 0.145, *p* = 0.181). However, the PCA plot in Figure 2 illustrates potential changes in bacterial composition across treatment phases, particularly in the S1 group, which appears to cluster separately from the intervention groups. Nevertheless, these visual trends were not supported by statistical significance (PERMANOVA, MRPP), suggesting that the observed changes may not be consistent or substantial. Future studies using larger sample sizes and additional beta diversity metrics are needed to confirm these findings and further evaluate the impact of antimicrobial interventions on the root canal microbiome.

Our analysis of antimicrobial interventions in primary endodontic infections revealed *Firmicutes* to be the dominant phylum across all stages of therapy. This result is consistent with the findings of Kruly et al. [28], who found *Firmicutes* to be the dominant phylum in the residual bacteriome in primary and secondary endodontic infections. Two recent studies investigating the effects of chemomechanical preparation and antimicrobial interventions on the root canal microbiome reported differing results: Alquria et al. [36] observed an increase in *Firmicutes* numbers, while de Kruly et al. [28] reported a reduction. Our findings align with those of de Kruly et al. [28], further supporting the notion that chemomechanical preparation can alter microbial community structures by reducing *Firmicutes* abundance. In the human digestive tract, *Firmicutes* and *Bacteroidetes* are the dominant phyla, and a higher *Firmicutes/Bacteroidetes* ratio indicates an out-of-balance and pro-inflammatory microbiome [37]. In the samples of Groups 2 and 4, there was a notable increase in *Bacteroidetes* rates (from 16.42% to 21.44% and from 18.81% to 24.24%) and a decrease in *Firmicutes* rates (from 45.64% to 42.11% and from 42.74% to 38.74%) following chemomechanical preparation. In a recent study [38], an increased *Firmicutes/Bacteroidetes* ratio was found in patients with marginal periodontitis. Based on these results, considering that marginal periodontitis and apical periodontitis are biofilm-induced conditions that cause disease with the same pathobiological mechanisms, it can be said that antimicrobial interventions during root canal procedures can lower the *Firmicutes/Bacteroidetes* ratio and promote a healthier and more harmonious root canal microbiome.

According to our results, the levels of *Bacillus*, *Bacteriodes*, *Listeria*, *Prevotella*, and *Faecalibacterium*, which are present in proportions above 3% in the root canal microbiome, remained mostly the same or had even increased after the disinfection stages of the root canal, providing evidence of their resistance to the antimicrobial treatments typically used. Most of these genera live in anaerobic or facultative anaerobic conditions. Sodium hypochlorite (NaOCl), as the primary irrigant used in root canal treatment, possesses proven antimicrobial and tissue-dissolving efficacy. However, it also presents significant limitations. Key among these are its high toxicity, which can cause severe tissue damage if it is extruded beyond the root canal, and potential to induce allergic reactions. A recent systematic review has highlighted these risks, emphasizing the importance of precise handling to minimize adverse effects [39]. Beyond NaOCl, it is essential to explore and develop complementary irrigants capable of eliminating anaerobic bacteria through active oxygen metabolites while also effectively reaching complex parts of the apical anatomy and maintaining a low risk of adverse effects.

In this study, we utilized LEfSe analysis to identify taxa that showed differing levels of abundance across all six taxonomic levels while following the main disinfection steps of a two-visit RCT. The statistical method used in LEfSe analysis has three stages, enabling the detection of significant differences, even with a small sample size. The use of linear discriminant analysis scores allows for the identification of biomarker taxa specific to groups within a biological context [40]. In the initially infected group (S1), there was a marked increase in the presence of *Candidatus Phytoplasma* witches-broom (Gram-positive), *Chryseobacterium_s* (Gram-negative/aerobic), *Arthrobacter_s* (Gram-positive/aerobic), *Tistrella mobilis* (Gram-negative/aerobic) and *Veillonella_s* (Gram-negative/anaerobic) in comparison to the other groups. These taxa can be considered typical inhabitants of initial root canal infections and could have been affected by the antimicrobial treatments used in RCT.

According to the LEfSe-based species-level results, one of the most remarkable findings of our study was the statistically significant higher abundance of *Bifidobacterium longum* (*B. longum*) (Gram-positive/anaerobic) species in the S3 samples after intracanal calcium hydroxide application. *B. longum* is a well-known probiotic strain residing in the gastrointestinal tract that promotes host well-being by modulating inflammatory cytokine levels, reducing levels of pro-inflammatory cytokines, and increasing anti-inflammatory cytokine levels through its metabolites [41,42,43]. Our results suggest that the niche adaptation of *B. longum* was enhanced in root canals disinfected with calcium hydroxide, offering new perspectives on its potential use as a probiotic in endodontic treatments. Its ability to prevent microbial dysbiosis and inhibit the growth of pathogenic microorganisms, as demonstrated in the gut [44,45], could similarly contribute to maintaining microbiome balance in root canals. Specifically, the capacity of *B. longum* to inhibit pathogenic bacterial colonization via capsular polysaccharides [46] may provide significant advantages by forming a protective barrier on root canal walls, thereby promoting infection control. Nevertheless, while LEfSe is a widely accepted method for identifying biomarkers, its results should be interpreted with caution, particularly in studies with small sample sizes or datasets exhibiting high variability. Validation with larger, independent datasets is therefore recommended to ensure the robustness of these findings and confirm their clinical relevance.

While the pH modulation induced by calcium hydroxide initially elevates the pH to approximately 12, its application in root canals for 1–2 weeks results in gradual stabilization to a range between 8 and 9.5 [47,48] during the S3 sampling phase. This range is higher than the optimal growth conditions for *B. longum* (5.0–7.2) [49]; however, the observed relative increase in its abundance may be attributed to other ecological factors within the root canal, such as the suppression of pathogenic bacteria by calcium hydroxide and the availability of ecological niches that indirectly support *B. longum*. This highlights the complexity of microbial interactions in the root canal environment and suggests that factors beyond pH modulation, including interspecies interactions and resource availability, play a significant role in shaping microbiome composition.

In addition, the S4 group exhibited a significantly higher level of *Dorea formicigenerans* (Gram-positive/anaerobic) following the final irrigation than the other groups in this study. In a recent study [28], the residual bacteriome was analyzed in teeth with primary and secondary infections after chemomechanical preparation, and taxa resistant to the RCT protocol were identified for both infection types. In line with our study, in the cited study, the authors also identified Gram-positive anaerobic species as the prevailing bacteria in root canals after antimicrobial procedures.

One of the limitations of this study is the inability to distinguish between viable and non-viable bacteria remaining in the root canal using 16S rRNA-based techniques. Incorporating dead bacteria could be beneficial for profiling the microbial community in defining the microbiome of an infection. However, it is disadvantageous to assess microbiome modulation when exposed to mechanical and chemical antimicrobial interventions. This may lead to an overestimation of the number of bacteria in post-treatment samples [50]. Specifically, the higher counts of ASVs observed between the S3 and S4 samples might have resulted from spotting gene remnants of non-living bacteria. The use of different final shaping instruments (R40 or R50) was associated with minor variations in the initial apical diameters of the distal canals in mandibular molars.While this approach was used to replicate clinical conditions, we acknowledge that variations in instrument size and canal anatomy could influence microbial disruption and tissue removal, potentially affecting the observed microbiome changes. Future studies with larger samples could explore this effect further.

One critical drawback of this investigation is the limited number of participants involved. The test power was calculated as 46.57% based on the current sample size (3 teeth in each group), whereas a minimum of 26 teeth per group would be required to achieve 80% power [26]. Despite these limitations, the sequencing depth achieved in this study averaged over 2000 reads per sample, ensuring sufficient coverage for microbial composition analysis. Nonetheless, the results should be interpreted within the context of these limitations, including the restricted sample size and resource constraints. Additionally, radiographic evaluations were performed by two calibrated observers, with a Cohen’s Kappa score of 0.85, ensuring inter-observer agreement. However, we acknowledge that the inclusion of a larger and independently validated observer group could further enhance the reliability of the findings and minimize potential bias.

The inability of paper point sampling to encompass the entire root canal microbiome might be another constraint of this study, since sampling from the lateral canals, isthmuses, ramifications, and deep dentin layers other than the main canal is not achievable using this method [51]. However, cryogenic grinding, as an alternative to paper point sampling, is not preferred for in vivo studies because it causes sample destruction [51]. In this study, descriptive hypervariable V3–V4 regions on the 16S rRNA genes of bacteria were analyzed as an alternative to whole-gene reading. A similarity range of 98.5%–99% in the 16S rRNA gene sequence is widely used to identify oral bacterial species [52]. High-quality sequencing results are achieved by sequencing from the V3–V4 hypervariable region [53], yet the species-level data obtained may lack full reliability [54]. Ultimately, because of the need for repeated sampling, this is a preliminary study with a small number of volunteers. We anticipate that this research will open up opportunities for upcoming research on modifying the root canal microbiome in endodontic procedures. Another limitation of this study is its focus on distal canals of mandibular molars. While this choice was made to standardize the sampling process and ensure methodological consistency, this focus may not fully represent the microbial diversity of other root canals or tooth types. Mandibular molars, with their relatively larger root canals, provide better access for reliable sampling; however, future studies involving multiple roots and a broader range of tooth types are necessary to obtain a more comprehensive understanding of the root canal microbiome.

Endodontic infections are polymicrobial, and microbial compositions can vary greatly among individuals [55,56]. RCT procedures involving physical and chemical disinfection can reduce the number of bacteria in root canals, but they cannot eliminate them completely [57]. Certain bacteria might not respond to standard root canal disinfection protocols. This study examined how the microbiome was modified at the end of the main phases of a two-visit RCT and determined whether the bacterial taxa were susceptible or resilient to the disinfection protocols. The identification of taxa resistant to standard disinfection protocols can provide valuable information for the development of next-generation disinfectants and antimicrobial procedures and enhance our comprehension of which disinfection methods are more effective for eliminating harmful pathogens in the root canal microbiome.

## 5. Conclusions

The results of this study demonstrate that a two-visit endodontic procedure carried out on teeth affected by primary apical periodontitis significantly altered the root canal microbiome. Chemomechanical preparation and intracanal calcium hydroxide medication reduced microbial diversity and highlighted resistant bacterial taxa. Additionally, the observed improvement in the *Firmicutes*/*Bacteroidetes* balance suggests that these interventions may contribute to fostering a microbiome with reduced pathogenic potential. The rise in *B. longum* numbers during the intracanal medication phase may not be solely due to pH conditions; rather, it could be linked to ecological transformations within the root canal, such as the inhibition of pathogenic bacteria. These findings underscore the complexity of microbial interactions in root canals and suggest that calcium hydroxide may support beneficial microbial shifts under certain conditions.

Further research is needed to explore these microbial changes and their clinical relevance, particularly the potential role of beneficial bacteria and oxygen byproducts in enhancing root canal treatment outcomes.

## Figures and Tables

**Table 1:** The total number of ASVs identified at the end of each antimicrobial phase.

Taxonomy	Groups			
	b(CMP)	a(CMP)	a(ICM)	a(FF)
Phylum	31	27	26	30
Class	74	68	59	68
Order	109	107	93	105
Family	153	145	139	156
Genus	252	208	212	232
Species	158	134	133	142
Total ASVs	777	689	662	733

Abbreviations: b(CMP)—before chemomechanical preparation, a(CMP)—after chemomechanical preparation, a(ICM)—after intracanal medication, and a(FF)—after final flush.

**Table 2:** The genera present at more than 1% abundance in the root canal microbiome post-antimicrobial treatment.

Genus	(Gram Staining/Oxygen Requirement)	b(CMP) %	a(CMP) %	a(ICM) %	a(FF) %
*Bacillus*	(Gr +/aerobic or facultative anaerobic)	4.66	5.08	4.78	4.5
*Bacteriodes*	(Gr −/anaerobic)	5.26	8.56	7.78	8.13
*Enterococcus*	(Gr +/aerobic or facultative anaerobic)	12.71	7.29	7.03	4.91
*Listeria*	(Gr +/facultative anaerobic)	7.72	7.62	9.58	8.02
*Prevotella*	(Gr −/anaerobic)	8.21	12.0	10.51	11.26
*Pseudomonas*	(Gr −/aerobic)	4.00	4.27	3.55	2.54
*Lactobacillus*	(Gr +/anaerobic)	2.80	3.86	2.34	2.21
*Lactococcus*	(Gr +/anaerobic)	2.61	1.97	2.55	1.68
*Blautia*	(Gr +/anaerobic)	1.50	1.79	1.77	1.69
*Candidatus cardinium*	(Gr −/anaerobic)	2.70	3.23	2.24	1.96
*Candidatus phytoplasma*	(^#^/aerobic)	1.82	1.06	4.76	5.19
*Clostridium*	(Gr +/anaerobic)	1.04	0.74	0.74	0.72
*Dialister*	(Gr −/anaerobic)	1.29	1.02	1.13	1.45
*Faecalibacterium*	(Gr −/anaerobic)	3.29	4.53	4.53	4.28
*Oscillospira*	(Gr +/anaerobic)	1.55	1.45	1.45	1.22
*Streptococcus*	(Gr +/facultative anaerobic)	2.47	0.54	1.29	1.10
*Acinetobacter*	(Gr −/aerobic)	2.11	1.32	0.84	1.29
*Ruminicoccus*	(Gr +/anaerobic)	0.74	1.12	0.92	0.87
*Succinivibrio*	(Gr −/anaerobic)	0.55	1.14	0.89	0.66
*Alicycliphilus*	(Gr −/facultative anaerobic)	0.02	0.05	*	1.14
*Coprococcus*	(Gr +/anaerobic)	0.66	0.91	0.91	1.11
*Ferruginibacter*	(Gr −/aerobic)	0.02	*	*	1.56
*Brevibacillus*	(Gr variable/aerobic)	0.29	0.54	1.17	0.07
*Niabella*	(Gr −/aerobic)	0.10	0.04	*	1.21
*Petrimonas*	(Gr −/anaerobic)	0.78	0.28	0.51	2.08
*Roseburia*	(Gr +/anaerobic)	0.90	1.04	1.18	1.15

* Below detection level. ^#^ Gram staining characteristic is unknown or not defined.

## Data Availability

The DNA-sequencing data generated and analyzed during the current study have been deposited in the NCBI SRA database under BioProject accession number PRJNA1173000. The data are now publicly available and can be accessed through the following link: https://www.ncbi.nlm.nih.gov/bioproject/PRJNA1173000 (accessed on 18 October 2024).
